# Active Control Loop of the BOROWIEC SLR Space Debris Tracking System

**DOI:** 10.3390/s22062231

**Published:** 2022-03-14

**Authors:** Tomasz Suchodolski

**Affiliations:** Centrum Badań Kosmicznych Polskiej Akademii Nauk (CBK PAN), Bartycka 18A, 00-716 Warszawa, Poland; suchodolski@cbk.poznan.pl

**Keywords:** Satellite Laser Ranging (SLR), space debris, active control loop, trajectory tracking, predictive control

## Abstract

The task of tracking cooperative satellites equipped with laser retroreflectors, by means of Satellite Laser Ranging (SLR), is an issue well described in the literature. The follow-up movement of the ground-based transceiver telescope behind an orbital object is based on the positional ephemeris data. The problem of controlling the follow-up motion of the telescope’s mount mostly in the Az/El configuration in this case boils down to the interpolation of the positional ephemeris data of the orbital object, which is the information input vector for the motion control system of the orthogonal and non-coupled axes of the propulsion system. In the case of tracking and determining the position of uncooperative objects (not equipped with retroreflectors), for which we can include rocket bodies and fragmentary elements, the task of keeping track of them becomes complex. The positional uncertainty of the ephemeris of uncooperative objects obtained mainly by means of survey radar acquisition requires the use of innovative solutions and complex control systems that enable the effective implementation of the tracking process. This paper presents innovative methods for the active control loop used in the SLR technique, consisting of dynamic motion corrections based on the passive optical acquisition with object recognition and analysis of the photon trace scattered from an orbital object.

## 1. Introduction

The issue of laser ranging measurements of artificial Earth satellites dates back to 1964. With the launch of the Beacon Explorer (Beacon-B) satellite by the National Aeronautics and Space Administration (NASA), the first laser measurement of the distance between a ground station and an orbital object was carried out [1]. Over the years with the development of laser technology, optics, control and executive systems, the accuracy of laser range measurements on the ground station—satellite path has increased many times and currently ranges from 1 cm to 2 cm. The fact of the progress made in building time and frequency references is also not without significance (hydrogen masers, cesium fountains) [2], thanks to which it is possible to accurately and precisely locate a specific observation in the time domain. The greatest advantage of the laser technique is the direct and absolute measurement of the distance between the ground station and the orbital object (satellite, rocket bodies, space debris). These measurements make a huge contribution to research on the implementation and definition of the terrestrial reference system, the International Terrestrial Reference Frame (ITRF), pole movement, the coordinates of the geocenter of the Earth’s gravitational field, the coordinate and velocity changes of the position of ground SLR stations [3,4], and determining the position of objects orbiting Earth with centimeter accuracy. However, due to the continual migration of continents, this is a complex and multi-stage process (the position of the ground station changes). The key solution to the above is the use of passive geodynamic satellites such as LAGEOS [5] (orbit altitude ∼ 6000 km) or ETALON [6] (orbit altitude ∼ 19,000 km), whose location at the Medium Earth Orbit (MEO) ensures the stability of their orbit over time (no influence of atmospheric effects). This constancy enables the determination of the current position of a given SLR station and, consequently, means it is suitable for measurements of other orbital objects. The continuous and almost simultaneous carrying out of this process by all laser ground stations enables the determination of their position, as well as the determination of geodynamic satellites’ orbits. In classical terms, laser range measurements are carried out for orbits from the Low Earth Orbit (LEO) region (altitude from 200 km to 2000 km) to Geostationary Orbit (GEO).

Currently, due to the dynamic exploration of orbits (LEO to GEO) associated with the launch of new satellites for the needs of industry, science, and defense, there is a need for increased monitoring of the traffic of orbital objects and the deorbiting process of used components such as rocket bodies, satellite fragments, as well as entire satellites [7]. From the point of view of the laser measurements performed by ground stations, orbital objects can be classified as cooperative and uncooperative. Cooperative objects, mainly satellites, are equipped with special systems of cubic prisms (retroreflectors) mounted on the outer casing, whose task is the low-loss reflection of the laser beam emitted from the ground station. Uncooperative objects are therefore all other orbital objects that are not equipped with retroreflectors (satellites, rocket bodies, space debris). This fact forces the use of high-energy laser measurement pulses, which increase the probability of a photon event in the form of light scattering from the object and its subsequent detection. Laser ranging measurements of cooperative satellites in the region from 350 km to 40,000 km are currently not a challenge in terms of tracking, but ranging measurements of objects with uncertain orbits or subject to deorbiting (altitude from 100 km to 200 km) require the use of new solutions in the fields of mechanics, drives, and control algorithms, hence the need to develop new control algorithms and the multiplication of measurement techniques in order to maximize information about the object during the pass over the ground station [8,9,10]. The laser technique, whose task is to accurately and precisely measure the range between the SLR ground station and orbital object in the time domain, also allows determining the dynamics of an object (spin) using a picosecond laser source [11,12]. Passive optical acquisition allows the use of a telescope and digital cameras (CCD, CMOS, sCMOS) to determine the orbital parameters of an object [13] and can also be used to obtain additional information using techniques such as light curves [14], which show the momentary variability of the brightness of objects, or further attempts to characterize the mission. The combination of these techniques makes it possible to obtain more data during a single pass of an object over a ground station.

The positional uncertainty of radar ephemerides in the form of Two-Line Elements set files (TLEs), in terms of their initial accuracy resulting from the inaccuracy of the radar survey technique, often cause problems in the tracking process behind an orbital object. Particular mention should be made of deorbiting events (orbit perigee $h_{p}<$ 200 km), the trajectory of which is constantly disturbed, implying a rapid obsolescence of ephemeris information, hindering the process of effective tracking of such objects. SLR stations perform point measurements, so the quality of the reflection of the theoretical ephemeris position of the orbital object above the station in relation to the real trajectory is important. Classically, the Field of View (FoV) of the transceiver telescope together with the photon detector enables tracking of an orbital object from the LEO region with an offset to the theoretical trajectory usually no greater than 50 ms (TimeBias). For offsets greater than the limit value of the FoV of the telescope, measurements are difficult to perform (the object is not visible in the field of view of the receiving telescope). In the case of regular tracking of cooperative satellites under the International Laser Ranging Service (mainly altimetric satellites and GNSS), it is possible to obtain current information about the appropriate correction to the object’s position (TimeBias). Thus, taking into account the value of the given offset, it is possible to correct the tracking ephemeris. In the case of tracking of uncooperative objects, the information about the theoretical trajectory, which is based on the TLE ephemeris, it is impossible to determine the offset of the position of the orbital object before performing the tracking process. This situation is particularly important in the case of re-entry events (object deorbiting), where the difference between the theoretical position of the object and the real trajectory is even a few seconds. It should also be mentioned that the positional accuracy of the TLE ephemeris over time is lost. For the LEO region, the TLE ephemeris loses its operational accuracy for the classic SLR station after 24 h. Due to the above, there is a need to make ongoing corrections of the tracking process behind the orbital object.

## 2. Satellite Laser Ranging Process

**Figure 1:**
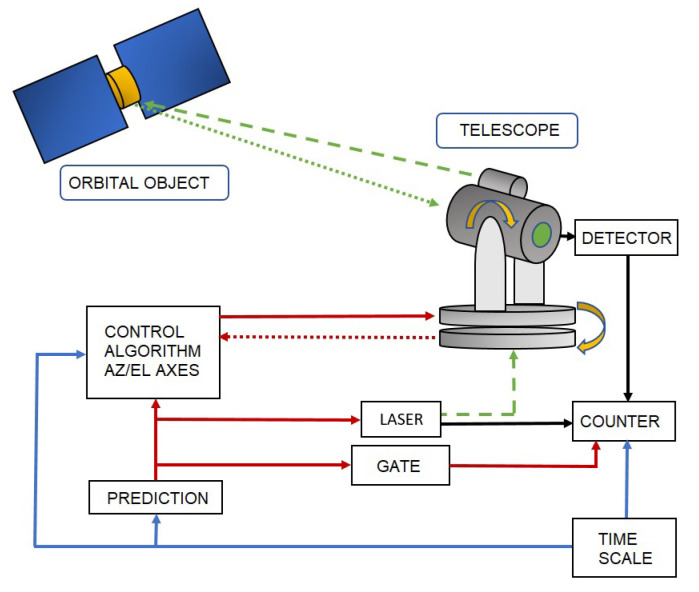
Block diagram of the SLR tracking system.

The laser ranging measurements of an orbital object are carried out by using a pulsed laser source, as a measuring beam emitter with the specified beam divergence $\theta \in (5^{\prime \prime },80^{\prime \prime })$, and a photon detector placed in the main focus of the receiving telescope operating mostly in the Az/El configuration (Figure 1). The detector is responsible for the acquisition of photons ($E_{x}\left(t\right)$) scattered by the orbital object (κ) from the previously emitted laser beam. The laser range measurement is based on the emission of a laser pulse of known length (x*1 ps, x*1 ns) at the $t_{START}$ moment of time, in the direction of the orbital object where some of the photons are scattered from the object’s surface. The $t_{STOP}$ moment of time, when the returning scattered photons are acquired by the photon detector, ends the measuring procedure. Therefore, the range measurement procedure consists of measuring the time interval $\Delta t_{p}$, which is necessary for the light pulse to travel the station–object–station distance ($\Delta t_{p}=t_{STOP}-t_{START}$). The task of the telescope’s movement control is to transform the ephemeris information $\zeta_{\kappa }$ about the position of the tracked object κ into the follow-up movement of the telescope’s mount. Therefore, the position of the spatial point $X_{\kappa }\left(t\right)$, $Y_{\kappa }\left(t\right)$, $Z_{\kappa }\left(t\right)$ should be transformed into the azimuth and elevation angles (Az(t), El(t)) for a given location of the SLR station. Due to the non-stationary nature of the process, the task is dynamic. Thus, the accuracy of the tracking process (mapping the motion of the telescope’s mount) directly depends on the control process and the accuracy of the drive system and intermediate mechanics [15].

### 2.1. Tracking Process

Taking into account the dynamics of the changes in the angular position (Az(t), El(t)) of the orbital object in relation to the ground station position in the time domain (mainly for the LEO region) and the required high accuracy of the tracking process (x*10arcsec; due to laser beam divergence) with respect to the uncertain $\sigma_{\kappa }$ of the theoretical position of the object, the task of the movement control should be carried out as continuously as possible (close to linear mapping). Therefore, the real object trajectory should be reflected by the iterative operation of the control algorithm, the result of which is projected onto the telescope follow-up movement. The iterative nature of the algorithm in predicting the successive points of the trajectory determines the computational time necessary to minimize the tracking error. In practice, it is assumed that the iteration interval should not be greater than 100 ms (mainly due to the high dynamics of position changes for objects from the LEO region). The tracking task is performed in two steps. In the first step, the time interval $<pass_{start}-pass_{stop}>$ should be estimated, in which the position of the object κ (in relation to the station) will enable its observation. Specifying the beginning of the observation time ($t_{0}$), the position of the object ($X_{t_{0}}$, $Y_{t_{0}}$, $Z_{t_{0}}$⇒$Az_{t_{0}}$/$El_{t_{0}}$), and the telescope angles ($Az_{T_{t_{0}}}$/$El_{T_{t_{0}}}$), the telescope is positioned relative to the beginning of the observation. This part of the procedure is not sensitive to the tracking process flow. The second stage starts at the time $t_{1}>t_{0}$, when the algorithm starts the tracking procedure.

The procedure is based on the implementation of a software control loop, which consists of the following activities:

- Importing the telescope’s axes encoder values: $Az_{t_{n}}^{\prime }/El_{t_{n}}^{\prime }$, in the $t_{n}$ moment of time;
- Prediction of the theoretical position of the object based on the ephemeris: $X_{t_{n}+1}$, $Y_{t_{n}+1}$, $Z_{t_{n}+1}$⇒$Az_{t_{n}+1}$/$El_{t_{n}+1}$, in the $t_{n+1}$ moment of time;
- Determining the difference in the form of a correction (ΔAz, ΔEl);
- Setting new motion parameters for the hardware layer ($V_{AZ},\;V_{EL}\Rightarrow v_{\alpha },\;v_{\beta }$).

**Figure 2:**
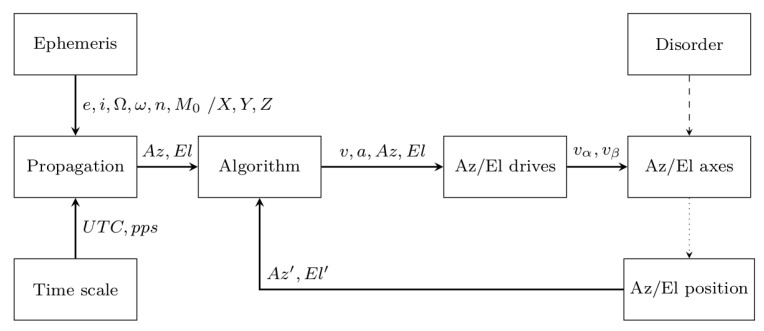
Block diagram of the SLR tracking control algorithm.

In contrast to the control loop implemented directly at the hardware layer, this approach allows flexibility in changing the motion parameters, although it is costly with additional software calculations outside the servomotor driver. There are a few approaches that determine the control method. The management software may provide with some advance ($t_{j:j\ll i}$) the drive controller information only about the new positions $X_{i}$, $Y_{i}$, $Z_{i}$ ($Az_{i}$/$El_{i}$) for a certain time $t_{i}$ (the controller must be equipped with a local RT clock). Then, the hardware layer, in the form of the NC servo controller, makes appropriate calculations/corrections (e.g., position, velocity, acceleration) so that the controlled axis follows the determined setting of the controlled parameters (Az/El) and in the moment of time $t_{i}$ reaches the position consistent with $X_{i}$, $Y_{i}$, $Z_{i}$ ($Az_{i}$/$El_{i}$). In this way, the entire adjustment process is performed locally by the drive controller (the data transmission of the determined new parameters between the management software and the drive controller does not affect the quality of the control process due to the predictive nature of these parameters). It is also possible to transfer to the hardware layer the new values of the position${}_{i}$, speed${}_{i}$, and acceleration${}_{i}$, developed by the management software in pseudo-real-time based on the position${}_{j:j\ll i}$, speed${}_{j:j\ll i}$, and acceleration${}_{j:j\ll i}$ (Az${}_{j:j\ll i}$/$El_{j:j\ll i}$) values received at time *t*${}_{j:j\ll i}$ from the drive controller for the previous designated position X${}_{j:j\ll i}$, Y${}_{j:j\ll i}$, Z${}_{j:j\ll i}$ (Figure 2).

The advantage of this solution is full control over the control process, but the disadvantage is the required pseudo-real-time data exchange between the managing software and the drive controller. The data processing chain in this case is obligated to make software transformations in specific time intervals (Δt∼ 100 ms), directly by correcting the drive control settings for the approaching moment of time $t_{i}$. Therefore, the algorithm at time *t*${}_{j:j\ll i}$ must estimate the new position of *X*${}_{i}$, *Y*${}_{i}$, *Z*${}_{i}$ ($Az_{i}$/$El_{i}$), establish the current state of the drives (e.g., position${}_{j:j\ll i}$, speed${}_{j:j\ll i}$, acceleration${}_{j:j\ll i}$), correct these parameters based on the required trajectory, and finally, send the newly estimated parameters as settings to the telescope’s drive controller. As mentioned, effective process control imposes the need to make calculations in time intervals Δt∼ 100 ms, including the time needed to transmit new motion parameters to the drive controller. It follows that designing a data processing algorithm requires defining time restrictions that determine the quality of the control loop operation.

### 2.2. Ephemeris Uncertainty

The tracking process behind an orbital object is based on a predictive model. The theoretical trajectory is predicted on the basis of the ephemeris (forecast) containing archival information obtained during previous positional measurements of the object in the time domain. In connection with the above, the quality of the reflection of the follow-up movement of the telescope in relation to the theoretical trajectory of the orbital object depends on the accuracy of the ephemeris data. Ephemeris data are obtained based on:

- Laser acquisition (distance to the object in the time domain, with centimeter accuracy);
- Passive optical acquisition (the angular position of the object in the time domain, with arcsec accuracy);
- Radar acquisition (distance and/or the angular position of the object in the time domain).

**Figure 3:**
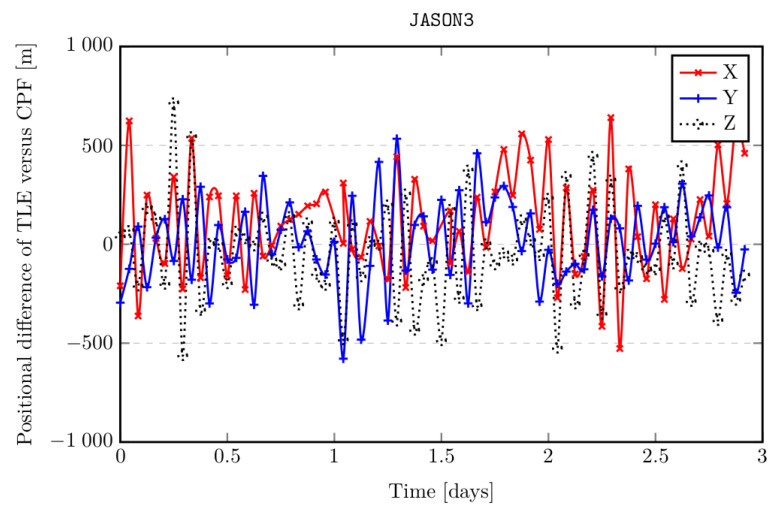
Positional uncertainty of the TLE data format relative to the CPF format using the SGP4 propagator (JASON3 satellite).

It should be emphasized that measurements of the position of an orbital object are performed in two ways: point-based (tracking behind a given orbital object ⇒ high resolution, high measuring accuracy) and in survey mode (wide-angle measurement of a given part of the sky ⇒ low resolution, low measurement accuracy, possible number of simultaneously tracked objects ≫ 1). In the case of laser measurements of cooperative satellites, ephemerides are created based on archival data obtained by the laser stations in the form of Consolidated Prediction Format files (CPF). This ephemeris contains information about the spatial position of the object (X, Y, Z) in the time domain. Thus, the task of tracking an object comes down to interpolating the ephemeris vector of the positions of the object in the time domain and transforming the interpolated values to the telescope’s reference frame (Az/El). However, in most cases, ephemerides are created on the basis of radar measurements (survey mode) and are made available in the form of TLE ephemerides with significant positional uncertainty. The TLE ephemeris for any moment of time $t_{n}$ is calculated on the basis of the given orbit parameters together with their initial epoch $t_{0}$, therefore, unlike the ephemeris of the CPF type, software calculations are necessary to determine the values of X, Y, Z. The uncertainty of the orbit predicted on the basis of the TLE ephemeris compared to the orbit interpolated from the ephemeris of the CPF type is shown in Figure 3.

**Figure 4:**
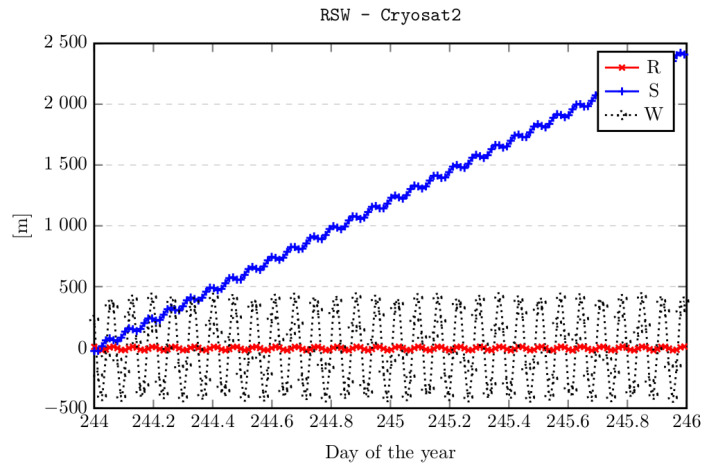
Timeliness of ephemeris data in the orbital object coordinate system (CryoSat-2 satellite).

Here, attention should be paid to the change in the quality of ephemeris over time. In the case of objects from the LEO region, the orbit parameters change due to the influence of the atmosphere and gravity (Figure 4) [16]. Therefore, to properly track an orbital object, it is necessary to use the current ephemeris data.

### 2.3. Laser Beam Propagation

**Figure 5:**
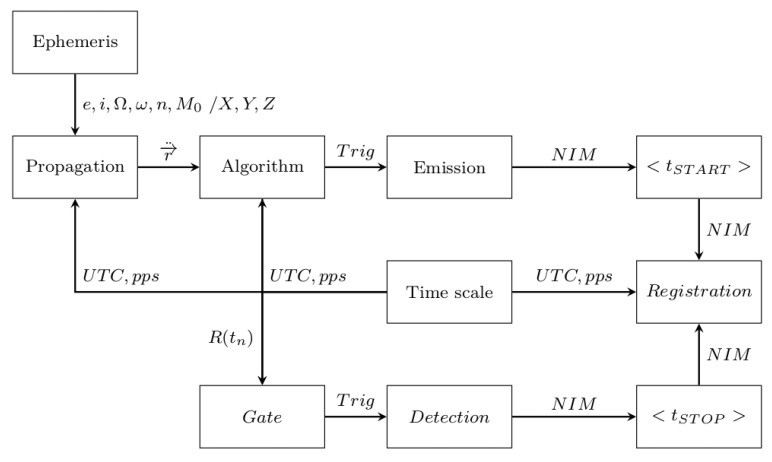
Block diagram of the laser ranging process.

An issue directly related to the laser measurement of the distance to an orbital object is the geometric shape of the laser beam. The laser distance measurement is based on the emission of a laser pulse towards the orbital object, where some of the photons are scattered from the object’s surface. The moment of time when the returning photons are acquired in the photon detector ends the measurement procedure (Figure 5).

As described in the Introduction, orbital objects subject to the range measurements by the SLR stations are divided into cooperative and uncooperative ones. Cooperative targets are equipped with retroreflectors mounted on the outer casing. A special feature of the solution is the ability to reflect the laser beam towards the point of its emission. Accordingly, there is no need to generate a high-energy laser beam in order to obtain a photon event caused by scattering from the measured object. Uncooperative targets are therefore all other orbital objects that are not equipped with retroreflectors. Therefore, in order to achieve photon events from an uncooperative orbital object, high-energy laser sources and highly sensitive photon detectors must be used [17,18].

#### 2.3.1. Beam Divergence

Each pulsed laser source is defined by the physical parameters, among which we distinguish: pulse energy (μJ, mJ), pulse width (ps, ns), repetition (Hz, kHz), beam divergence (mrad). With reference to Degnan’s radar link equation (Equation (1)) [19], the probability of registering a photon scattered from an orbital object strongly depends on the energy of the laser pulse and laser beam divergence. This dependence is as follows:

(1) $$n_{pe}=\eta_{q}\left(E_{T}\frac{\lambda }{hc}\right)\eta_{t}G_{t}\sigma {\left(\frac{1}{4\pi R^{2}}\right)}^{2}A_{r}\eta_{r}T_{a}^{2}T_{c}^{2},$$

where $\eta_{q}$ is the detector quantum efficiency, $E_{T}$ is the laser pulse energy, λ is the laser wavelength, *h* is Planck’s constant, *c* is the velocity of light in a vacuum, $\eta_{t}$ is the transmit optics’ efficiency, $G_{t}$ is the transmit gain, σ is the orbital object cross-section, *R* is the slant range to the object, $A_{r}$ is the effective area of the telescope receive aperture, $\eta_{r}$ is the efficiency of the receive optics, $T_{a}$ is the one-way atmospheric transmission, and $T_{c}$ is the one-way transmissivity of cirrus clouds (when present). The transmitter gain $G_{t}$ of the system with the use of a Gaussian laser beam is defined as:

(2) $$G_{t}=\frac{8}{\theta_{t}^{2}}exp\left[-2{\left(\frac{\theta }{\theta_{t}}\right)}^{2}\right],$$

where $\theta_{t}$ is half of the divergence angle between the axis of the laser beam and the area of intensity 1/$e^{2}$ and θ is the error of the positioning uncertainty of the emitted beam.

The geometrical divergence defines the degree of enlargement of the cross-sectional area of the laser beam with the distance and is expressed in mrad. Laser measurements are made for orbital objects from the regions from LEO to GEO, so the distance between the SLR station and the orbital object ranges from about 350 km to 42,000 km. The aim of the measurement process is to propagate the laser beam between the ground station and the orbital object. The laser source emits a laser beam with given physical parameters and a known starting diameter, which is usually a few millimeters (<1 cm) in size. Taking into account the large distance separating the ground station and the orbital object (>350 km), the geometric divergence parameter of the beam determines its cross-section size on the orbit. Standard laser sources for SLR needs are characterized by a geometric divergence of the beam at the level of 0.5 mrad–1.0 mrad (1.0 mrad = 206.26 arcsec). Assuming a 1 cm diameter of the laser beam with a divergence of 1 mrad, for an orbital object distance of 350 km, the orbital beam’s cross-sectional area is 700 m (for a 42,000 km distance, it is 84 km). This fact is of no great importance when tracking of cooperative objects with low-energy laser emission sources. The energy density that is deposited at a given orbital altitude in this case is not critical. However, in the case of tracking of uncooperative objects in order to obtain a photon event in the form of scattering a portion of light from the object and its subsequent detection, a high energy density deposited on the object’s surface is required. Therefore, it forces the use of systems correcting the divergence of the laser beam. Such a system is classically composed of a set of lenses for which the distance relation between them affects the final beam divergence. In this way, it is possible to correct the divergence parameter to a minimum value of $\theta \in (1^{\prime \prime },5^{\prime \prime })$. For example, when using the divergence correction system, assuming a 1 cm diameter of the output laser beam with a divergence of 0.02 mrad, for a distance of the orbital object of 350 km, the cross-sectional size of the beam on the orbit is 14 m (for 42,000 km, it is 1.6 km, respectively). Such a divergence value guarantees a high energy density, which is necessary to acquire a detected photon event from an uncooperative object. However, such a divergence value combined with the ephemeris uncertainty described in Section 2.2 may lead to acquisition difficulties. The relatively small cross-section of the laser beam on the orbit in the form of the spot size and an ephemeris with high uncertainty may prevent effective spot measurement of the object.

#### 2.3.2. Coordination of Beam Emission

The classic SLR system consists of a receiving telescope, mostly in the Az/El configuration, with a photon detector and a coaxially mounted laser beam emitter with a divergence corrector (Figure 1). Depending on the type of the laser, it is possible to mount the laser source directly on the telescope housing or, in the case of the scientific lasers with a picosecond pulse width, there is a need to propagate the laser beam from an air-conditioned room, using the Coudé path with intermediary mirrors. Each solution ensures the alignment of the telescope axis with the laser beam emitter. Unfortunately, the imperfection of the ephemerides described in Section 2.2 and the impact of laser beam divergence on the effectiveness of the measurement described in Section 2.3.1 requires the correction of the telescope’s movement in order to frame the object. Ephemeris uncertainty is mainly related to the time shift along the object’s trajectory (along-track) and is determined by the TimeBias parameter. In the case of cooperative satellites, this parameter is determined by other SLR stations during previous measurements. In this way, it is possible to make an appropriate time correction before starting the tracking process. A large number of uncooperative objects makes it impossible to determine the value of this parameter. In such a case, a movable prismatic head in the *XY* plane system can be used behind the laser source output. This solution enables manual or automatic angular offset of the laser beam in relation to the telescope’s axes. Laser measurement only determines the distance to the object in the time domain; therefore, the angular offset of the laser beam does not cause disturbances to the results.

## 3. Active Control Loop

### 3.1. Passive Optical Acquisition Active Control Loop

**Figure 6:**
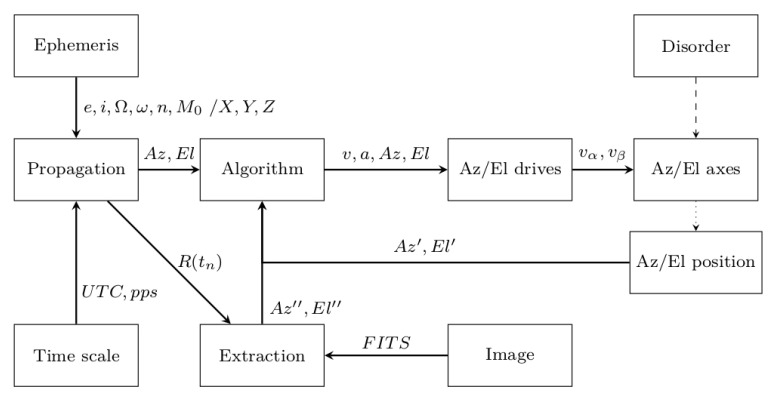
Block diagram of the active control correction algorithm based on ephemeris data and image analysis.

Due to the positional uncertainty of the TLE ephemeris (Section 2.2) used to track space objects, an appropriate algorithm was developed and implemented, combining the passive optical acquisition technique with the process of correcting the telescope motion parameters (Section 2.1) [20]. In the case of objects from the LEO region (h < 2000 km), due to the relatively small distance between the SLR station and the orbital object, it is possible to determine the position of an orbital object illuminated by the Sun (day/night terminator) using the wide-angle photographic acquisition technique (a digital camera with an additional telescope). The information obtained in the form of images can be transformed into the real coordinates of the orbital object ($Az^{\prime \prime }/El^{\prime \prime }$), in relation to the ephemeris position (Az/El). The use of high-speed planetary cameras, which are characterized by a short exposure time (0.001 s–1 s) and fast data transfer (*x* * MB/s), leads to obtaining information about the position of the object in close to real-time. Using postprocessing, it is possible to extract the position of the object from the image frame and relate the vector of position changes to the current movement of the telescope (Figure 6).

It should be mentioned that passive optical acquisition also enables the reference of the object’s position to the recorded star trails (astrometry) [21]. Such information can therefore be treated as a correction of the theoretical ephemeris position, which is introduced into the control loop of the Az/El axes of the telescope mount. A more accurate method of active control correction is carried out by analyzing the images obtained from the main focus of the receiving telescope, which is characterized by a smaller field of view, resulting from the large focal length of the instrument. For this purpose, the receiving optical path, whose main task is to detect photons scattered by the orbital object from the previously emitted laser beam, shares this with a digital camera. Due to the simultaneous emission of the laser beam (532 nm—green or 1064 nm—infrared), it is impossible to acquire an image without the use of an appropriate method of optical signal filtering. For this purpose, a dichroic optical filter is used, which splits the orthogonally detected signal in two directions. The split point is chosen so as to separate the optical signal of the laser beam from the optical path of the camera. For laser sources operating at 532 nm, a split point above or below this value is applied. In this way, two signal-separated optical bands are obtained. In the case of laser sources operating at a wavelength of 1064 nm, a split point is used in the range of 900 nm–1000 nm. Then, the entire visible spectrum can be used for photographic acquisition using a digital camera. The method due to the large focal length of the telescope used (main mirror diameter over 50 cm) requires the use of digital cameras equipped with large matrices (e.g., 50 mm × 50 mm) to increase the image detection area. On the other hand, the relatively small field of view of the camera together with the telescope determines the control loop intervals below the standard value of 100 ms.

### 3.2. Photon Event Active Control Loop

**Figure 7:**
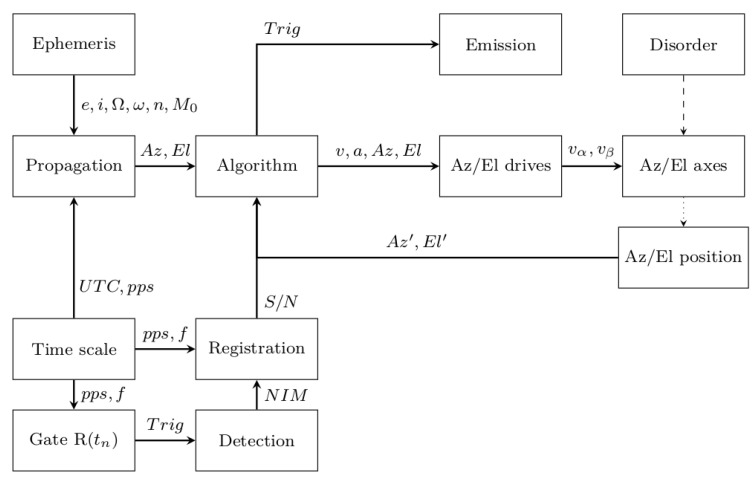
Block diagram of the active control correction algorithm based on ephemeris data and photon events.

The active control correction of the telescope’s movement behind the orbital object according to the proposed algorithm can also be made by quantifying the photon trace scattered from the orbital object. In this case, the data stream of the photon event recorder obtained during the measurement process in close to real-time is analyzed (Figure 7). The absence or negligible number of photon events $E_{n}$ at the expected time $t_{n}$, which is determined by the distance $R_{x}$ to the orbital object, forces the procedure of searching for the object to be performed.

In the case of uncooperative space debris, the quantitative measures of acquired photon events are completely different from the cooperative ones. Due to the lack of a retroreflector element, the number of returning photons is incomparably low. This does not change the fact that each return echo from space debris objects provides information about their orbit and of the behavior of these objects (e.g., spin). Furthermore, the notion of a normal point in the case of uncooperative space debris becomes less relevant and even negligible. Space debris mainly consists of unstable objects, so any information is valuable. Therefore, the algorithm provides two alternative paths for assessing the presence of photon events: by the comparison of the similarity of successive photon events in the time domain or by evaluating the return rate. Both paths depends on the end capabilities of the laser–detector pair in terms of the maximal detection range and given Radar Cross-Section (RCS) of the population of the orbital objects.

The advantage of this solution is the ability to correct mount motion parameters even when the object is invisible (daylight) or the object is at higher orbits (MEO, GTO), which makes passive optical acquisition with a short exposure time difficult to perform. The use of such an algorithm leads to the automation of the measurement process, which in the case of 24/7 operation relieves the staff of the SLR station. There is no need for the operator to manually search for an object, even for laser ephemerides (CPF), which always have some TimeBias. Another convenience compared to the manual search for an object by the operator is that the algorithm works based on the distance parameter $R_{x}$ and the beam divergence $\theta_{y}$ for a given moment of time $t_{n}$. In manual mode, the operator sees the emission spot of the laser beam on the preview screen, but does not know its real cross-section at the orbital altitude. From this, it follows that its manual *X*/*Y* or manual spiral search can create search gaps. Another aspect is that the operator is unable to calculate for a given moment of time the exact time interval that is needed to obtain an echo from the object (long time intervals for the MEO and GEO regimes). Thus, the operator may wait too short or too long to receive an echo, which reduces the effectiveness of the measurement. The operation of the algorithm takes into account all these components; therefore, it leads to faster and more effective measurements. The main disadvantage of the process is the time it takes to complete the procedure. The most common approach is the spiral search method, which iteratively increments the Az/El axes’ deflection. With reference to Section 2.3.1, a spiral radius *r* calculation is implemented depending on the laser beam divergence used. Hereby, an optimal search of the area is ensured, which leads to a quick search for an object, eliminating the possibility of omitting the object.

## 4. Experiment

### 4.1. Test Platform

**Figure 8:**
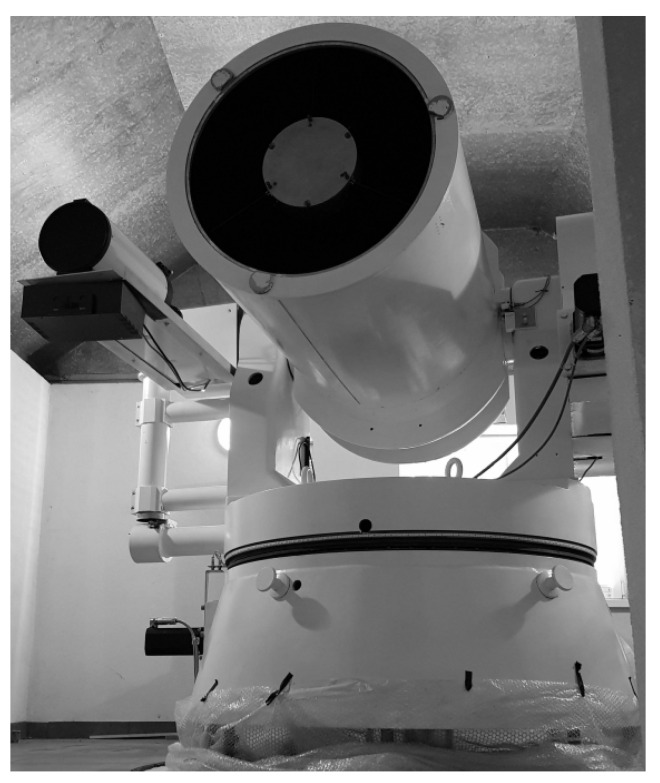
Cassegrain telescope in the Az/El configuration—hybrid laser system.

The main development task was to launch the second and inactive SLR tracking system in the form of a telescope with a main mirror diameter of 65 cm. This telescope is a tube that has been mounted on an existing non-instrumented horizontal mount (Figure 8). The sensor evaluation process consisted of the development and implementation of all system components, including: the control chain, actuators in the form of mechanics and drives, and the implementation of the logic layer in the form of the control and data processing algorithms. These works included all elementary activities, from the design of the mounting and coupling elements, to programming the servo drive systems with appropriate operating characteristics. Due to the required high accuracy of the tracking process of orbital objects, amounting to x* 10”, with the simultaneous operating speed $V_{x}<20^{\circ }$/s and reposition speed $V_{R}∼40^{\circ }$/s, the task was to develop an appropriate control algorithm for such a complex system. Carrying out a process that is strictly established in the time domain forced choosing such a system platform that allows proceeding in near real-time (Linux). The task consisted of program calculations, the acquisition and processing of the signals, the process of motion control, as well as the physical implementation of this process in the form of telescope follow-up movement behind an orbital object in the time domain. The multiple paths and complexity of the issue required the use of a complete tool system in the form of software that was suitable for the supervision and implementation of the above tasks. The software was developed in C# using the MySQL database with interfaces for PLC and servo drives.

### 4.2. Active Control Loop Tests

**Figure 9:**
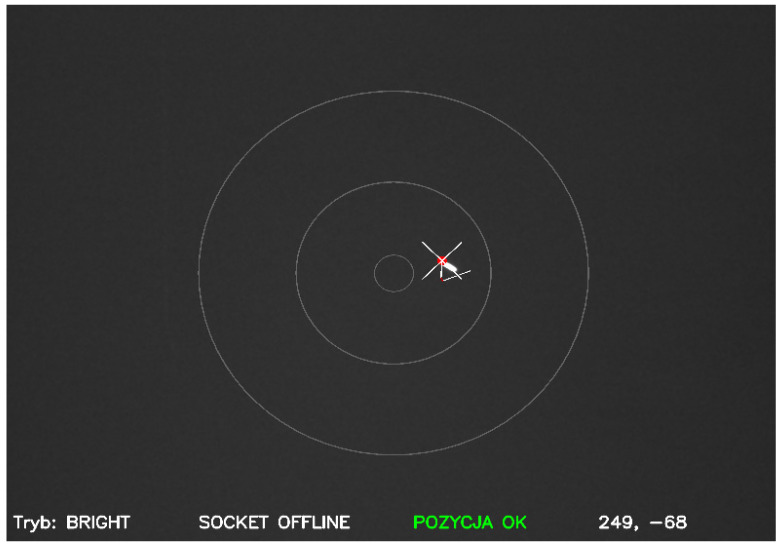
Object extraction and position determination (OpenCV).

The system was equipped with an additional RC8${}^{\prime \prime }$ telescope, which enabled image acquisition for active control loop. A planetary-type camera with a USB3 interface was selected, which provided images for further processing. Knowing the physical parameters of the CMOS sensor in the form of the matrix size and pixel size, in combination with the telescope parameters, it was possible to determine the field of view of such a set and its optical resolution. The selected ASI 1600MMC camera provided a field of view of 0.63${}^{\circ }$× 0.48${}^{\circ }$ (37.83${}^{\prime }$× 28.59${}^{\prime }$). This value made it possible to carry out an active control loop test using the extraction of the object’s position from the image frame and the determination of its positional offset in relation to the telescope axes. The OpenCV package was used for image analysis. By analyzing successive image frames, the object’s position offset expressed in pixels in relation to the origin of the coordinate plane was determined, which is the intersection of the *X* and *Y* axes (Figure 9).

By comparing the positional offset with respect to the field of view of the instrument, it was possible to calculate the actual angular shift ($\Delta Az^{\prime \prime }\left(t_{n}\right),\Delta El^{\prime \prime }(t_{n}$)) of the object κ relative to the telescope axes ($x_{AZ}$ = 0, $y_{EL}$ = 0). In accordance with Section 2.1, the calculated positional offset had to be included in the axis motion control algorithm (Figure 6), according to the procedure:

(3) $$TLE_{\kappa }\left(t_{n+1}\right)\Rightarrow Az\left(t_{n+1}\right)+\Delta Az^{\prime \prime }\left(t_{n}\right),El\left(t_{n+1}\right)+\Delta El^{\prime \prime }\left(t_{n}\right),$$

where $Az(t_{n+1})$ and $El(t_{n+1})$ are the theoretical angle values of the object κ in the next iteration step and $\Delta Az^{\prime \prime }\left(t_{n}\right)$ and $\Delta El^{\prime \prime }\left(t_{n}\right)$ are the angle values of the current angular offset of the object κ. In the practical implementation, the control software sent to the servo drivers the velocity values ($V_{Az}$, $V_{El}$) defined as:

(4) $$V_{Az}=\left[\left(Az\left(t_{n+1}\right)-Az^{\prime }\left(t_{n}\right)\right)+\Delta Az^{\prime \prime }\left(t_{n}\right)\right]*\gamma_{AZ},$$

(5) $$V_{El}=\left[\left(El\left(t_{n+1}\right)-El^{\prime }\left(t_{n}\right)\right)+\Delta El^{\prime \prime }\left(t_{n}\right)\right]*\gamma_{EL},$$

where $Az(t_{n+1})$ and $El(t_{n+1})$ are the theoretical angle values of the object κ in the next iteration step, $Az^{\prime }\left(t_{n}\right)$ and $El^{\prime }\left(t_{n}\right)$ are the values of the real angles from the axes’ encoders, $\Delta Az^{\prime \prime }\left(t_{n}\right)$ and $\Delta El^{\prime \prime }\left(t_{n}\right)$ are the angle values of the current angular offset of the object κ, and $\gamma_{AZ}$ and $\gamma_{EL}$ are parameters that define the dependence of the mechanical transmission ratio per time unit. It should be noted that a limitation of the minimum offset value was defined, which may affect the performance of the correction. Therefore, only significant corrections to the telescope’s position were made, preventing the continuous operation of the algorithm. There was also a need to define the field of view of the photon detector in connection with the main telescope as an active detection area.

The determined positional offset can also be used independently to correct the laser beam emission angle, which was programmatically tested. In such a case, a movable prismatic head in the *XY* plane described in Section 2.3.2 was controlled by a value of $Az^{\prime \prime }\left(t_{n}\right),El^{\prime \prime }\left(t_{n}\right)$.

### 4.3. Photon Event Active Control Loop

**Figure 10:**
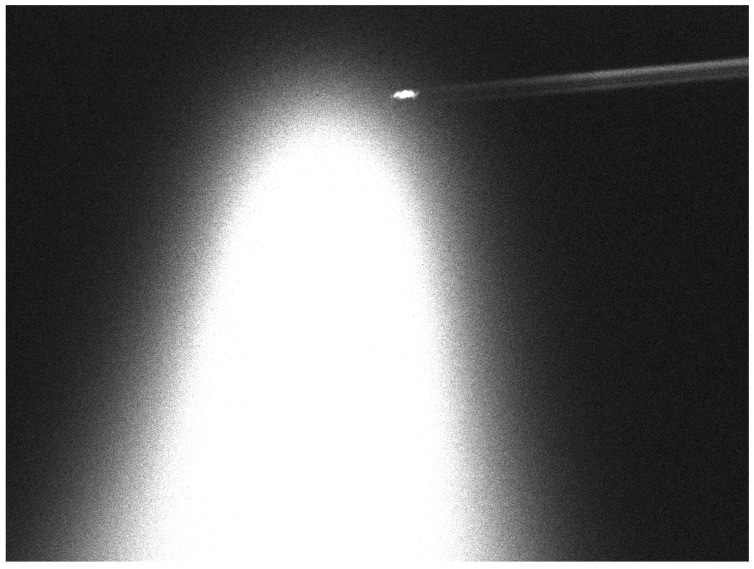
Laser tracking of the CryoSat-2 satellite using two SLR systems at CBK PAN.

Due to the unavailability of a scientific laser source and a photon detector, the photon event active control loop described in Section 3.2 was partially simulated. For this purpose, a demonstration CW laser was used to simulate the emission of a pulsed scientific laser. The use of a laser beam divergence corrector and a photon event dataset were also simulated numerically. An illuminated object from the LEO region in the form of the CryoSat-2 satellite was intentionally selected for the experiment. Figure 10 shows the object tracking process with the simultaneous use of two SLR systems located at the SRC PAS Borowiec Astrogeodynamic Observatory.

**Figure 11:**
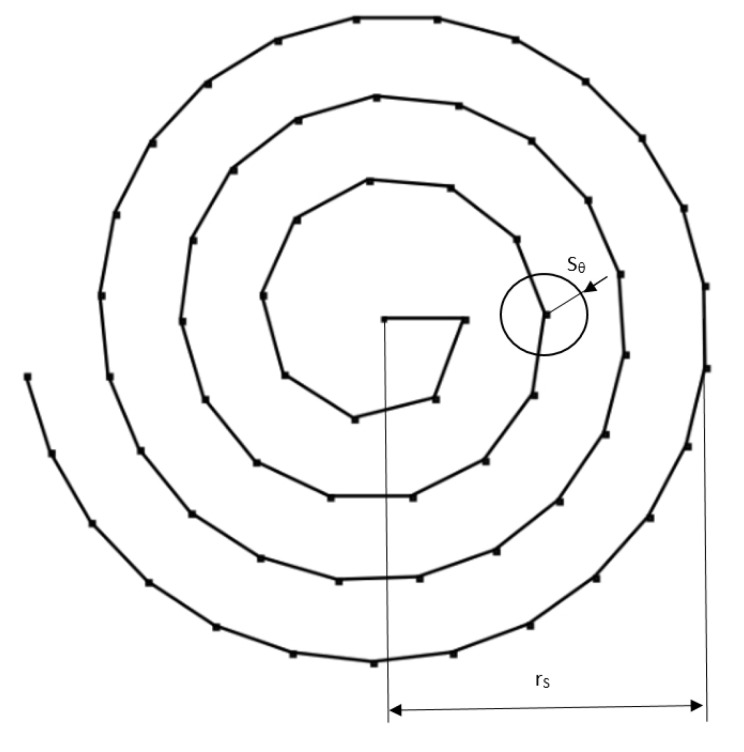
Correction of the telescope axis motion based on the modified Archimedean spiral.

The control algorithm uses the properties of the Archimedean spiral defined by the formula in polar coordinates (r,φ):

(6) r=a+b*φ,

where *a* moves the center point of the spiral outward from the origin (positive *a* toward φ=0, negative *a* toward φ=π), while *b* controls the distance between spiral loops. It follows from the above that the position of the next spiral point is proportional to the angle φ over time. Using this dependence, it was possible to make a modification consisting of taking into account the value of the beam divergence (laser spot size for a given altitude) and the radius ($r_{S}$) of the search area of the orbital object κ. In this way, it was possible to define the number of loops depending on the search area and the degree of coverage by the laser spot size. Such a dependency in the local polar coordinate system $R^{2}$ can be described as:

(7) $$X=X_{0}\left(Az_{\kappa }\left(t_{n}\right)\right)+cos\left(\varphi +R\right)*\left[\varphi *\frac{r_{S}}{\left(\frac{r_{S}}{2\pi *S_{\theta }}\right)}\right]$$

(8) $$Y=Y_{0}\left(El_{\kappa }\left(t_{n}\right)\right)+sin\left(\varphi +R\right)*\left[\varphi *\frac{r_{S}}{\left(\frac{r_{S}}{2\pi *S_{\theta }}\right)}\right]$$

where $X_{0},\;Y_{0}$ are the origin of the coordinate system, $Az_{\kappa }\left(t_{n}\right),El_{\kappa }\left(t_{n}\right)$ are the theoretical angle values of the object κ in the $t_{n}$ moment of time, φ is the the processed angle of the spiral, R is the possible rotation of the spiral, $r_{S}$ is the search area radius, and $S_{\theta }$ is the spot size radius at the orbital altitude. Each successive iteration of the algorithm is dependent on an offset proportional to the spot size at the orbital altitude. The steps of the algorithm also depend on the time interval $\Delta t_{R_{\kappa }}$, which is necessary to obtain the photon trace ($E_{x}$) scattered from the orbital object. The operation of the algorithm was interrupted when a photon event was detected, according to the procedure described in Section 3.2. The operation of such a modified algorithm is shown in Figure 11.

Using the above modification, it was possible to define the search area radius ($r_{S}$) of the object κ depending on the $\zeta_{\kappa }$ ephemeris uncertainty expressed in arcmin. The scaling of the number of loops and the angle offset were performed automatically according to Formulas (7) and (8), so it is possible to use this solution regardless of the orbital region knowing only the distance to the orbital object and the laser beam divergence. The advantage of this solution is the duality of using the algorithm to correct the telescope’s movement during the object tracking process or to control of movable prismatic head in the *XY* plane system, which can be used behind the laser source.

## 5. Conclusions

The process of the laser ranging measurement of orbital objects with the uncertain ephemerides is a difficult issue. Besides, the growing amount of space debris forces the use of new solutions to maximize information about the orbits of such objects. The available number of laser tracking sensors is too small to improve the quality of the orbits obtained from radar survey sensors. This paper proposed solutions to improve the effectiveness of measuring the distance to the orbital objects with uncertain orbits using the active control loop implemented at the Borowiec SLR station. The presented algorithms for the active correction of mount motion control and automatic object search functionality led to an increase in the effectiveness of measuring objects such as space debris. Cooperative objects are only a small subset of the space debris population, so these objects should be treated as uncooperative targets in terms of the measurements based on the TLE ephemeris. An additional advantage of these solutions is the ability to perform measurements in automatic mode, which with 24/7 operations relieves the need for the SLR station operator. Further research is needed to optimize the proposed solutions, which depend on the type of object and the orbital regime. Depending on the capabilities of a given laser station, it is possible to carry out detailed analyses with the implementation of the described algorithms. The proposed analyses may refer to the detection of the coordinate offset values of a specific orbital object on the basis of information stored in the database. Such information can be gathered from previous observations of an orbital object using the photon event active control loop or passive optical acquisition active control loop algorithms. The obtained and averaged value of the positional offset stored in the database may be the starting point to perform a new observation of the given orbital object. The analysis can be made taking into account the type of ephemerides (TLE, CPF), the orbital altitude, as well as the suitability of ephemeris use depending on the epoch of its generation. Another aspect that can be investigated is the analysis of the required beam divergence in order to find the object in the initial phase. Such an analysis can be performed for object types (e.g., rocket bodies), as well as individually for a specific orbital object. Furthermore, a quantitative statistic of photon events for a specific object can be made. In this way, it will be possible to determine the effectiveness of subsequent observations during the target search process. A separate issue is the parameterization of algorithms, which depends on the specific configuration of the laser sensor. The parameters of the receiving telescope, including the photon detector, and the telescope mount motion control loop are of importance here. Commissioning of the second SLR system at Borowiec Astrogeodynamic Observatory based on a dedicated laser and photon detector will allow for many analyses taking into account the impact of ephemeris uncertainty on the reaction time of the SLR system and the obtained results. Optimizing the operation of algorithms during daytime observations is not without significance, which will be the next stage of the research. The effectiveness of the proposed solutions can be verified only in the conditions of the availability of a measuring laser and a photon detector. Only in this way, by obtaining the reflections from the orbital object, will it be possible to determine the effectiveness of the operating algorithms.

## Data Availability

Not applicable.
